# A proper protocol for routine ^18^F-FDG uEXPLORER total-body PET/CT scans

**DOI:** 10.1186/s40658-023-00573-4

**Published:** 2023-09-11

**Authors:** Huiran Hu, Yanchao Huang, Hongyan Sun, Kemin Zhou, Li Jiang, Jinmei Zhong, Li Chen, Lijuan Wang, Yanjiang Han, Hubing Wu

**Affiliations:** 1 Nanfang PET Center, Nanfang Hospital, Southern Medical University, 1838 Guangzhou Avenue North, Guangzhou, 510515 Guangdong Province People’s Republic of China; 2 United Imaging Healthcare, Shanghai, People’s Republic of China

**Keywords:** ^18^F-FDG, uEXPLORER total-body PET/CT, Image quality, Scan time, Injected radioactivity, Reconstruction algorithm

## Abstract

**Background:**

Conventional clinical PET scanners typically have an axial field of view (AFOV) of 15–30 cm, resulting in limited coverage and relatively low photon detection efficiency. Taking advantage of the development of long-axial PET/CT, the uEXPLORER PET/CT scanner with an axial coverage of 194 cm increases the effective count rate by approximately 40 times compared to that of conventional PET scanners. Ordered subset expectation maximization (OSEM) is the most widely used iterative algorithm in PET. The major drawback of OSEM is that the iteration process must be stopped before convergence to avoid image degradation due to excessive noise. A new Bayesian penalized-likelihood iterative PET reconstruction, named HYPER iterative, was developed and is now available on the uEXPLORER total-body PET/CT, which incorporates a noise control component by using a penalty function in each iteration and finds the maximum likelihood solution through repeated iterations. To date, its impact on lesion visibility in patients with a full injected dose or half injected dose is unclear. The goal of this study was to determine a proper protocol for routine ^18^F-FDG uEXPLORER total-body PET/CT scans.

**Results:**

The uEXPLORER total-body PET/CT images reconstructed using both OSEM and HYPER iterative algorithms of 20 tumour patients were retrospectively reviewed. The quality of the 5 min PET image was excellent (score 5) for all of the dose and reconstruction methods. Using the HYPER iterative method, the PET images reached excellent quality at 1 min with full-dose PET and at 2 min with half-dose PET. The PET image reached a similar excellent quality at 2 min with a full dose and at 3 min with a half dose using OSEM. The noise in the OSEM reconstruction was higher than that in the HYPER iterative. Compared to OSEM, the HYPER iterative had a slightly higher SUVmax and TBR of the lesions for large positive lesions (≥ 2 cm) (SUVmax: up to 9.03% higher in full dose and up to 12.52% higher in half dose; TBR: up to 8.69% higher in full dose and up to 23.39% higher in half dose). For small positive lesions (≤ 10 mm), the HYPER iterative had an obviously higher SUVmax and TBR of the lesions (SUVmax: up to 45.21% higher in full dose and up to 74.96% higher in half dose; TBR: up to 44.91% higher in full dose and up to 93.73% higher in half dose).

**Conclusions:**

A 1 min scan with a full dose and a 2 min scan with a half dose are optimal for clinical diagnosis using the HYPER iterative and 2 min and 3 min for OSEM. For quantification of the small lesions, HYPER iterative reconstruction is preferred.

**Supplementary Information:**

The online version contains supplementary material available at 10.1186/s40658-023-00573-4.

## Introduction

Positron emission tomography/computed tomography (PET/CT) is a non-invasive imaging modality for diagnosis, staging, treatment evaluation and prognosis prediction of malignant diseases [1–6]. It also plays an important role in the diagnosis of cardiovascular and neurological diseases [7, 8]. ^18^F-Fluorodeoxyglucose (^18^F-FDG), as the most widely used tracer, can provide important information, such as tumour glycolysis in lesions, which can be used to reflect the proliferative activity of tumours [9, 10].

Conventional clinical PET scanners typically have an axial field of view (AFOV) of 15–30 cm [11], resulting in limited coverage and relatively low photon detection efficiency. In addition, a whole-body image requires multiple (6–8) bed position acquisitions [12, 13]. Taking advantage of the development of long-axial PET/CT, the uEXPLORER PET/CT scanner (uEXPLORER, United Imaging Medical Technology Co., Ltd., Shanghai, China) [14, 15] with an axial coverage of 194 cm increases the effective count rate by approximately 40 times compared to conventional PET scanners [16, 17], which makes fast PET acquisition for the total body possible [18, 19].

Currently, statistical iterative reconstruction methods are the most widely used image reconstruction methods, and the ordered subset expectation maximization (OSEM) algorithm is the gold standard. OSEM algorithms approach the acquired image by successive updated approximations, which are repeated until the difference between the projections of the reconstructed image and the actual recorded image falls below a specific level. The major drawback of OSEM is that the iteration process must be stopped before convergence to avoid image degradation due to excessive noise [20], which reduces the reconstruction accuracy and lesion contrast [21].

A new Bayesian penalized-likelihood iterative PET reconstruction, named HYPER iterative, was developed and is now available on the uEXPLORER total-body (TB) PET/CT. The HYPER iterative incorporates a noise control component by using a penalty function in each iteration and it finds the maximum likelihood solution through repeated iterations. This penalty function acts as a noise suppression term and is controlled by a penalization factor (termed regularization intensity) [22–24]. It was reported by Haojun Yu et al. that the lesion visibility scores were significantly higher in HYPER iterative reconstructions than in OSEM (*P* < 0.05) in a study with ultralow ^18^F-FDG activity on TB PET/CT scans [25]. However, its impact on quantification and contrast in patients with a full or half injected dose is still unclear.

Although an expert consensus on oncological ^18^F-FDG total-body PET/CT imaging (version 1) has currently been proposed, the whole procedure was based on OSEM reconstruction instead of the HYPER iterative [26]. HYPER iterative reconstruction may introduce some changes in the procedure. Thus, the goal of this study was to determine an optimal protocol for routine ^18^F-FDG uEXPLORER total-body PET/CT scans.

## Materials and methods

### Patients

This retrospective study was approved by the Ethics Committee of Southern Medical University (No. NFEC-2022-515). Written informed consent was obtained from every patient before undergoing PET/CT. From April to July 2022, 20 patients underwent uEXPLORER total-body PET/CT scan for tumour staging. The patients were16 men and 4 women, aged from 34 to 73 years. Among them, 18 patients were diagnosed with lung cancer, one with oesophageal cancer and one with colon cancer. The diagnosis was confirmed by histopathology. All of the patients fasted for more than 6 h before the injection of ^18^F-FDG, and their fasting blood glucose was controlled within the normal range. Ten patients were injected with full-dose ^18^F-FDG (3.7 MBq/kg) (the full-dose group). Another 10 patients were injected with half-dose ^18^F-FDG (1.85 MBq/kg) (the half-dose group) according to the literature [18]. The PET data of above 20 patients were retrospectively reconstructed using both OSEM and HYPER iterative algorithms, and the images of PET were comparatively reviewed. The relevant clinical data of the enrolled patients are shown in Table 1. The procedure in this study was carried out according to the principles expressed in the Declaration of Helsinki.

**Table 1:** Clinical information of patients

Characteristic	Full dose group (*n* = 10)	Half dose group (*n* = 10)	*P*
Age (years)	60.6 ± 10.8 (range 38–73)	50.9 ± 10.9 (range 34–68)	0.971
*Sex*			0.264
Female	1	3	
Male	9	7	
Height (cm)	171.6 ± 7.2	163.1 ± 6.2	0.015
Weight (kg)	70.4 ± 11.8	59.9 ± 7.3	0.075
BMI (kg/m^2^)	23.8 ± 2.5	22.5 ± 2.7	0.631
Blood glucose level before injection (mmol/L)	5.7 ± 1.1	6.2 ± 1.0	0.123
Injected dose (MBq)	259.1 ± 45.5	118.6 ± 16.5	< 0.001
*Histopathology*			0.329
Lung cancer	8	10	
Oesophagus cancer	1	0	
Colorectal cancer	1	0	

### Total‑body PET/CT examination

Approximately 60 min after injection with ^18^F-FDG, the patients underwent TB PET/CT imaging scans. The TB PET/CT scan was performed using a single bed for 5 min. The entire 5 min 3D list-mode dataset was then split into 4 min, 3 min, 2 min, 1 min, 30 s and 10 s images to simulate different time acquisitions, and they were reconstructed using OSEM and HYPER iterative algorithms. The parameters for OSEM reconstruction were as follows: TOF and PSF modelling, 3 iterations and 20 subsets, matrix of 192 × 192, slice thickness of 2.886 mm, FOV 600 mm (pixel size 3.125 × 3.125 × 2.89 mm^3^) with a Gaussian postfilter (3 mm) and attenuation and scatter correction applied. The parameters for the HYPER iterative reconstruction were as follows: TOF and PSF modelling, regularization intensity 0.28, matrix of 192 × 192, slice thickness of 2.886 mm, FOV 600 mm, with attenuation and scatter correction applied, but no postfilter applied. The reconstructed pixel size, FOV and iteration number were predefined by the manufacturer of uEXPLORER, and they were not allowed to be changed by the users. Penalty strength β is the only adjustable parameter with a range of (0, 1). The CT scan parameters were set as follows: tube voltage 120 kV, tube current 140 mAs, pitch 1.0, collimation 0.5 mm and reconstructed slice thickness of 0.5 mm.

### Analysis of the PET/CT imaging

The subjective analysis of the image quality was visually assessed by four experienced nuclear medicine physicians independently (two for the full dose and two for the half dose), who were blinded to the HYPER iterative or OSEM reconstructions. A 5-point Likert scale was used to subjectively evaluate the image quality based on the following three perspectives: overall impression of the image quality, image noise and lesion visibility (1 = unacceptable image quality for diagnosis, 2 = acceptable image quality with no need to repeat the scan, 3 = fair image quality as in routine practice, 4 = good image quality with performance exceeding routine practice and 5 = excellent image quality) [27].

**Fig. 1:**
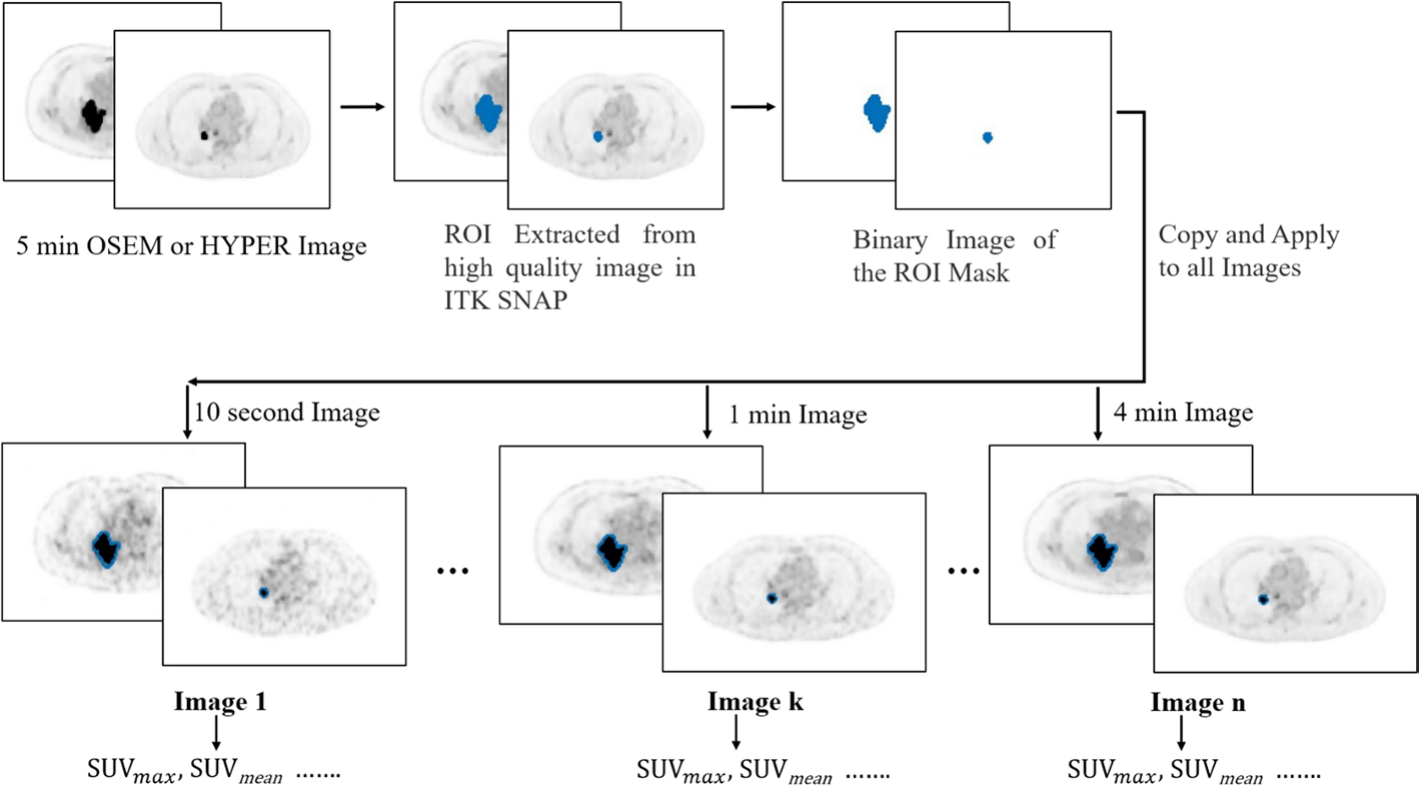
Flow chart to explain how the ROI was drawn in the image of 5 min on ITK-SNAP software and copied to the images of 10 s, 30 s, 1 min, 2 min, 3 min and 4 min to measure SUVmax, SUVmean, etc.

Quantitative evaluation of the image quality was performed by an experienced technician under the supervision of a nuclear medicine physician blinded to the actual images. To evaluate the uniformity of the distribution of radioactivity, a 2D circular region of interest (ROI) was drawn in a homogeneous area in the right liver lobe and in the ascending aortic arch as the blood pool using ITK-SNAP software, which was developed by Paul Yushkevich, PhD, of the Penn Image Computing and Science Laboratory (PICSL) at the University of Pennsylvania, and Guido Gerig, PhD, of the Scientific Computing and Imaging Institute (SCI) at the University of Utah. To avoid intrahepatic lesions and large blood vessels, the diameter of the ROI within the right liver lobe was limited to 2 cm. The maximum of the standard uptake value (SUVmax), the mean of the standard uptake value (SUVmean), and its standard deviation (SD) in the ROI were recorded. Lesions with a diameter of less than 10 mm were selected as small positive lesions, and those with a diameter of more than 2 cm were selected as large positive lesions. In ITK-SNAP, ROIs for lesions were originally drawn on the axial slice of the 5-min image reconstructed by OSEM or the HYPER iterative algorithm. Each ROI was manually drawn and automatically adapted to an ROI with an SUVmax of 40% contour, which was then copied to the images 10 s, 30 s, 1 min, 2 min, 3 min and 4 min to measure the SUVs in every ROI using MATLAB program written in-house. The 5-min image was used for drawing the ROIs because the lesion could be visualized clearly on it (Fig. 1). The tumour–background ratio (TBR) was defined by dividing the SUVmax of the lesion by the mean standardized uptake value of the background activity in the blood pool. The metabolic tumour volume (MTV) of lesions was measured using an isocontour threshold of 40% SUVmax. For the analysis of the homogeneity background, the ratio of the SD (*SD*_*OSEM*_*/SD*_*HYPER*_*)* and the difference between the two algorithms were calculated as follows:

$${\text{SD}}_{{{\text{OSEM}}}} /{\text{SD}}_{{{\text{HYPER}}}} = \frac{{{\text{SD}}_{{{\text{OSEM}}}} - {\text{SD}}_{{{\text{HYPER}}\;{\text{Iterative}}}} }}{{{\text{SD}}_{{{\text{HYPER}}\;{\text{Iterative}}}} }}$$

$${\text{Difference}} = \frac{{{\text{SUV}}_{{{\text{max}}\_{\text{OSEM}}}} - SUV_{{{\text{max}}\_{\text{HYPER}}\;{\text{Iterative}}}} }}{{{\text{SUV}}_{{{\text{max}}\_{\text{HYPER}}\;{\text{Iterative}}}} }}$$

The difference in the SUVmax for the positive large lesions and positive small lesions at each time point between the two algorithms is as follows:

$${\text{Difference}} = \frac{{{\text{SUV}}_{{{\text{max}}\_{\text{HYPER}}\;{\text{Iterative}}}} - {\text{SUV}}_{{{\text{max}}\_{\text{OSEM}}}} }}{{{\text{SUV}}_{{{\text{max}}\_{\text{OSEM}}}} }}$$

### Statistical analysis

Statistical analyses were performed with SPSS 24.0 software for Windows (IBM SPSS Inc., Armonk, NY, USA), and a* P* value < 0.05 was considered statistically significant. The interrater agreement of visual scores for image quality was tested with the weighted kappa test, and a kappa value > 0.81 was considered excellent agreement. The Wilcoxon signed-rank test was used to compare the scores and PET parameters between the HYPER Iterative and OSEM reconstruction algorithms with different scan times and injected doses.

## Results

### Subjective visual evaluation of the image quality

The inter-reader agreement for the image quality showed a kappa of 0.963 between the two readers for the full dose and a kappa of 0.990 between the two readers for the half dose.

**Fig. 2:**
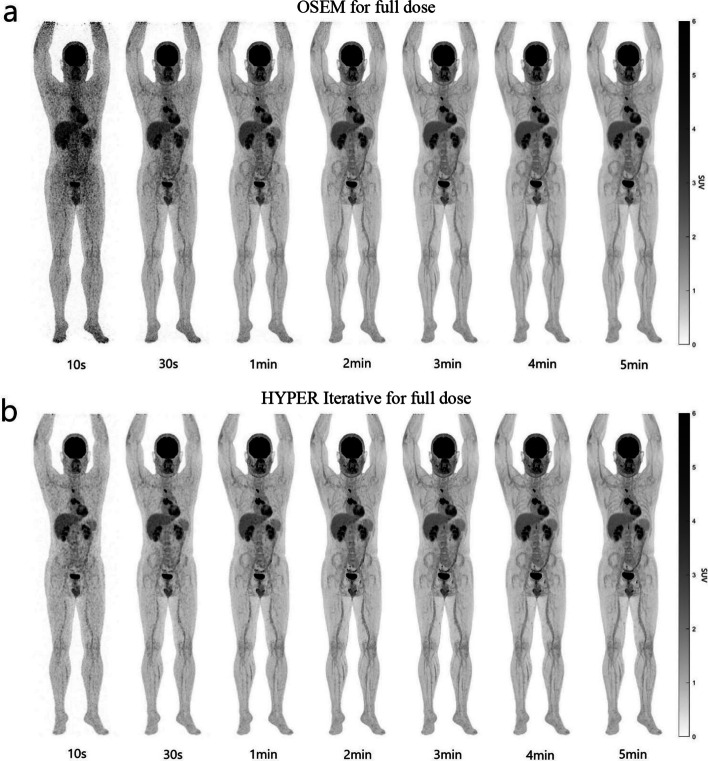
Total-body ^18^F-FDG PET MIP images of different scan times reconstructed by OSEM and HYPER Iterative in a patient with lung cancer injected with full dose ^18^F-FDG. **a** ^18^F-FDG PET MIP images of 10 s, 30 s, 1 min, 2 min, 3 min, 4 min and 5 min, respectively, reconstructed by OSEM. The quality of OSEM-10 s, OSEM-30 s and OSEM-1 min images was low due to low counting rate and large signal noise, especially the OSEM-10 s and OSEM-30 s. The OSEM-2 min image reached the high quality, either that of OSEM-3 min to OSEM-5 min. **b** ^18^F-FDG PET MIP images of 10 s, 30 s, 1 min, 2 min, 3 min, 4 min and 5 min, respectively, reconstructed by HYPER Iterative. The quality of HYPER Iterative-10 s and HYPER Iterative-30 s had lower signal noise and higher quality than the counterparts by OSEM although they were still not excellent. The HYPER Iterative-1 min image reached the high quality, either that of HYPER Iterative-2 min to HYPER Iterative-5 min

In the full-dose group, the OSEM-10 s, OSEM-30 s and OSEM-1 min images were noisy and characterized by roughness and poor homogeneity (Fig. 2), which were scored 1.00 ± 0.00, 1.85 ± 0.37 and 2.75 ± 0.44, respectively. These three groups could not meet the high-quality requirements for clinical diagnosis (Table 2). The image quality of OSEM-2 min reached a nearly excellent level, with a score of 4.80 ± 0. 42 (Fig. 2 and Table 2). As the scan time increased, the OSEM-3 min to OSEM-5 min images were all excellent (scores, 5.00 ± 0.00) (Fig. 2 and Table 2). For the HYPER Iterative, HYPER Iterative-10 s and HYPER Iterative-30 s had less noise and were better than their counterparts using OSEM (Fig. 2). The corresponding scores were 2.75 ± 0.44 and 4.05 ± 0.22, respectively, which were significantly higher than those of OSEM-10 s and OSEM-30 s (*P* < 0.05) (Table 2), but still did not reach the clinical diagnosis requirement. The PET image quality nearly reached an excellent level at 1 min, with a score of 4.90 ± 0.31 (Fig. 2 and Table 2). After that, the images of HYPER Iterative-2 min to HYPER Iterative-5 min were all excellent, with scores of 5.00 ± 0.00 (Fig. 2 and Table 2).

**Table 2:** Visual scores of PET images reconstructed by HYPER Iterative and OSEM at different scan times in the full-dose group

Time	Reconstruction algorithm	Reader 1					Reader 2					Average score	*P*
		Score 5	Score 4	Score 3	Score 2	Score 1	Score 5	Score 4	Score 3	Score 2	Score 1		
10 s	OSEM	0	0	0	0	10	0	0	0	0	10	1.00 ± 0.00	0.000
	HYPER Iterative	0	0	8	2	0	0	0	7	3	0	2.75 ± 0.44	
30 s	OSEM	0	0	0	9	1	0	0	0	8	2	1.85 ± 0.37	0.000
	HYPER Iterative	1	9	0	0	0	0	10	0	0	0	4.05 ± 0.22	
1 min	OSEM	0	0	8	2	0	0	0	7	3	0	2.75 ± 0.44	0.000
	HYPER Iterative	9	1	0	0	0	9	1	0	0	0	4.90 ± 0.32	
2 min	OSEM	8	2	0	0	0	8	2	0	0	0	4.80 ± 0.42	0.125
	HYPER Iterative	10	0	0	0	0	10	0	0	0	0	5.00 ± 0.00	
3 min	OSEM	10	0	0	0	0	10	0	0	0	0	5.00 ± 0.00	1.000
	HYPER Iterative	10	0	0	0	0	10	0	0	0	0	5.00 ± 0.00	
4 min	OSEM	10	0	0	0	0	10	0	0	0	0	5.00 ± 0.00	1.000
	HYPER Iterative	10	0	0	0	0	10	0	0	0	0	5.00 ± 0.00	
5 min	OSEM	10	0	0	0	0	10	0	0	0	0	5.00 ± 0.00	1.000
	HYPER Iterative	10	0	0	0	0	10	0	0	0	0	5.00 ± 0.00	

**Fig. 3:**
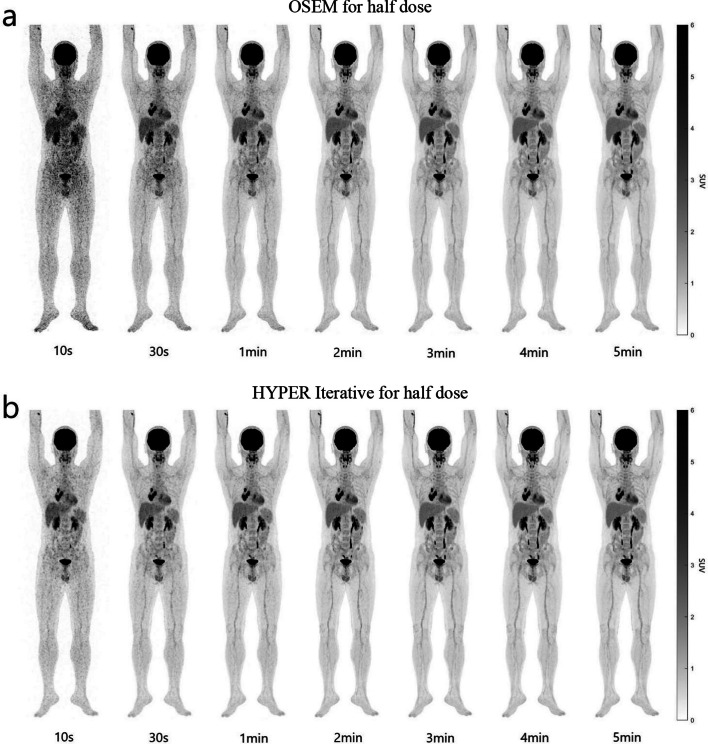
Total-body ^18^F-FDG PET MIP images of different scan times reconstructed by OSEM and HYPER Iterative in a patient with lung cancer injected with half dose^18^F-FDG. **a** ^18^F-FDG PET MIP images of 10 s, 30 s, 1 min, 2 min, 3 min, 4 min and 5 min, respectively, reconstructed by OSEM. The quality of OSEM-10 s, OSEM-30 s, OSEM-1 min and OSEM-2 min images was low due to low counting rate and large signal noise, especially the OSEM-10 s and OSEM-30 s. The OSEM-3 min image reached the high quality, either that of OSEM-4 min and OSEM-5 min. **b** ^18^F-FDG PET MIP images of 10 s, 30 s, 1 min, 2 min, 3 min, 4 min and 5 min, respectively, reconstructed by HYPER Iterative. The quality of HYPER Iterative-10 s, HYPER Iterative-30 s and HYPER Iterative-1 min had lower signal noise and higher quality than the counterparts by OSEM although they were still not excellent. The HYPER Iterative-2 min image reached the high quality, either that of HYPER Iterative-3 min to HYPER Iterative-5 min

In the half-dose group, OSEM-10 s, OSEM-30 s, OSEM-1 min and OSEM-2 min were worse than their counterparts in the full-dose group (Fig. 3), and their scores were 1.00 ± 0.00, 1.75 ± 0.44, 2.70 ± 0.47 and 3.75 ± 0.44, respectively (Table 3). The quality of the OSEM-3 min images nearly reached the excellent level, with a score of 4.85 ± 0.37. Both OSEM-4 min and OSEM-5 min images reached excellent scores of 5.00 ± 0.00. The images of HYPER Iterative-10 s, HYPER Iterative-30 s and HYPER Iterative-1 min were better in quality than those of their counterparts with OSEM. Their scores were 2.00 ± 0.00, 2.90 ± 0.31 and 3.75 ± 0.44, respectively, but they did not reach an excellent level. The image quality of PET reconstructed by HYPER Iterative reached an excellent level at 2 min with a score of 4.80 ± 0.41. The images from HYPER Iterative-3 min to HYPER Iterative-5 min were all excellent (score 5.00 ± 0.00).

**Table 3:** Visual scores of PET images reconstructed by HYPER Iterative and OSEM at different scan times in the half-dose group

Time	Reconstruction algorithm	Reader 1					Reader 2					Average score	*P*
		Score 5	Score 4	Score 3	Score 2	Score 1	Score 5	Score 4	Score 3	Score 2	Score 1		
10 s	OSEM	0	0	0	0	10	0	0	0	0	10	1.00 ± 0.00	0.000
	HYPER Iterative	0	0	0	10	0	0	0	0	10	0	2.00 ± 0.00	
30 s	OSEM	0	0	0	8	2	0	0	0	7	3	1.75 ± 0.44	0.000
	HYPER Iterative	0	0	10	0	0	0	0	8	2	0	2.90 ± 0.31	
1 min	OSEM	0	0	7	3	0	0	0	7	3	0	2.70 ± 0.47	0.000
	HYPER Iterative	0	8	2	0	0	0	7	3	0	0	3.75 ± 0.44	
2 min	OSEM	0	8	2	0	0	0	7	7	0	0	3.75 ± 0.44	0.000
	HYPER Iterative	8	2	0	0	0	8	2	0	0	0	4.80 ± 0.41	
3 min	OSEM	9	1	0	0	0	8	2	0	0	0	4.85 ± 0.37	0.250
	HYPER Iterative	10	0	0	0	0	10	0	0	0	0	5.00 ± 0.00	
4 min	OSEM	10	0	0	0	0	10	0	0	0	0	5.00 ± 0.00	1.000
	HYPER Iterative	10	0	0	0	0	10	0	0	0	0	5.00 ± 0.00	
5 min	OSEM	10	0	0	0	0	10	0	0	0	0	5.00 ± 0.00	1.000
	HYPER Iterative	10	0	0	0	0	10	0	0	0	0	5.00 ± 0.00	

### Quantitative evaluation of PET/CT image quality

#### The effect of the two reconstruction algorithms on the homogeneous radioactivity areas

In both the full-dose and half-dose groups, the shorter image acquisition time resulted in a larger SD in the liver and mediastinal blood pool for both algorithms. Compared to the HYPER Iterative reconstruction, OSEM reconstruction had a larger SD for each scan time from 10 s to 5 min (all *P* < 0.05), but the difference decreased as the acquisition time increased, as shown in Table 4. For the full-dose group, the SD by OSEM with a scan time of 10 s was up to 4–5 times higher than that with the HYPER Iterative (Table 4). The difference increased to approximately 8 times higher in the liver for the half-dose group with an acquisition time of 10 s (Table 4). The SDs generated by OSEM or HYPER Iterative were larger for the half dose than for the full dose with the same scan time.

**Table 4:** SD of radioactivity distribution in homogeneous background in the liver and blood pool in different scan times and injected dose reconstructed by HYPER iterative and OSEM

Homogeneous background	Time	Full dose group (*n* = 10)				Half dose group (*n* = 10)			
		HYPER Iterative	OSEM	SD_OSEM_/SD_HYPER_	*P*	HYPER Iterative	OSEM	SD_OSEM_/SD_HYPER_	*P*
Liver SD	10 s	0.11 ± 0.08	0.49 ± 0.38	3.50 ± 2.05	0.005	0.13 ± 0.11	1.17 ± 1.65	8.29 ± 7.00	0.005
	30 s	0.04 ± 0.04	0.11 ± 0.07	2.45 ± 1.51	0.005	0.06 ± 0.04	0.25 ± 0.13	3.48 ± 1.66	0.005
	1 min	0.02 ± 0.02	0.05 ± 0.03	1.40 ± 0.71	0.005	0.05 ± 0.03	0.14 ± 0.10	2.34 ± 1.37	0.005
	2 min	0.02 ± 0.01	0.04 ± 0.02	1.09 ± 0.47	0.005	0.02 ± 0.01	0.07 ± 0.03	1.95 ± 0.53	0.005
	3 min	0.02 ± 0.01	0.03 ± 0.02	0.87 ± 0.55	0.005	0.01 ± 0.01	0.03 ± 0.02	1.24 ± 0.54	0.005
	4 min	0.02 ± 0.01	0.03 ± 0.02	0.42 ± 0.34	0.007	0.01 ± 0.01	0.03 ± 0.01	1.03 ± 0.83	0.005
	5 min	0.01 ± 0.01	0.02 ± 0.01	0.29 ± 0.28	0.022	0.01 ± 0.01	0.02 ± 0.02	0.97 ± 0.54	0.005
Blood pool SD	10 s	0.07 ± 0.04	0.35 ± 0.20	4.84 ± 3.26	0.005	0.11 ± 0.06	0.54 ± 0.34	4.90 ± 1.67	0.005
	30 s	0.04 ± 0.05	0.14 ± 0.13	2.38 ± 1.20	0.005	0.04 ± 0.02	0.13 ± 0.07	2.52 ± 1.08	0.005
	1 min	0.03 ± 0.01	0.07 ± 0.03	1.32 ± 0.56	0.005	0.03 ± 0.02	0.09 ± 0.06	1.62 ± 1.00	0.005
	2 min	0.02 ± 0.02	0.04 ± 0.03	0.95 ± 0.41	0.005	0.02 ± 0.02	0.05 ± 0.03	1.48 ± 0.69	0.005
	3 min	0.01 ± 0.01	0.02 ± 0.02	0.92 ± 0.60	0.005	0.02 ± 0.01	0.04 ± 0.02	1.16 ± 0.45	0.005
	4 min	0.01 ± 0.01	0.02 ± 0.01	0.74 ± 0.50	0.005	0.02 ± 0.01	0.03 ± 0.02	0.91 ± 0.39	0.005
	5 min	0.01 ± 0.01	0.01 ± 0.01	0.74 ± 0.51	0.005	0.02 ± 0.01	0.03 ± 0.02	0.60 ± 0.20	0.005

The SUVmax of the blood pool and liver reconstructed by OSEM was significantly higher than that reconstructed by the HYPER Iterative for a scan time from 10 s to 2 min. A falsely higher SUVmax in the blood pool and liver homogeneous areas reconstructed by OSEM was observed for a scan time less than 2 min, which was approximately 28.43–33.97% higher for the full dose at 10 s and up to 42.01–49.68% higher for the half dose at 10 s (Table 5). For a scan time of 2–5 min, the SUVmax of the blood pool and the liver gradually decreased to a stable low level. Compared to the full dose, the falsely higher SUVmax in the blood pool reconstructed by OSEM was more obvious for the half dose (Table 5).

**Table 5:** SUVmax of radioactivity distribution in homogeneous background in the liver and blood pool in different scan times and injected dose reconstructed by HYPER Iterative and OSEM

Viscera	Time	Full dose group (*n* = 10)				Half dose group (*n* = 10)			
		HYPER Iterative	OSEM	Difference (%)	*P*	HYPER Iterative	OSEM	Difference (%)	*P*
Liver SUVmax	10 s	3.07 ± 0.44	3.91 ± 0.66	28.43 ± 20.45	0.005	2.78 ± 0.88	4.26 ± 2.31	49.68 ± 38.73	0.005
	30 s	2.79 ± 0.42	3.21 ± 0.42	15.47 ± 6.42	0.005	2.67 ± 0.58	3.18 ± 0.78	18.53 ± 9.18	0.005
	1 min	2.75 ± 0.44	2.90 ± 0.49	5.32 ± 2.76	0.007	2.50 ± 0.62	2.85 ± 0.80	13.39 ± 5.13	0.005
	2 min	2.67 ± 0.38	2.77 ± 0.38	4.07 ± 1.50	0.005	2.41 ± 0.55	2.60 ± 0.62	7.66 ± 2.41	0.005
	3 min	2.64 ± 0.36	2.72 ± 0.37	3.08 ± 1.42	0.005	2.36 ± 0.54	2.50 ± 0.58	6.21 ± 0.81	0.005
	4 min	2.68 ± 0.35	2.73 ± 0.37	1.99 ± 1.32	0.009	2.35 ± 0.54	2.47 ± 0.59	4.98 ± 1.94	0.005
	5 min	2.60 ± 0.31	2.64 ± 0.33	1.33 ± 1.29	0.017	2.31 ± 0.52	2.40 ± 0.56	3.74 ± 1.73	0.005
Blood pool SUVmax	10 s	2.34 ± 0.45	3.14 ± 0.89	33.97 ± 24.03	0.005	2.23 ± 0.50	3.18 ± 0.86	42.01 ± 19.67	0.005
	30 s	2.14 ± 0.42	2.62 ± 0.54	23.12 ± 16.11	0.005	2.00 ± 0.42	2.49 ± 0.61	25.94 ± 23.79	0.005
	1 min	2.00 ± 0.26	2.37 ± 0.35	18.78 ± 13.34	0.005	1.96 ± 0.48	2.29 ± 0.45	20.19 ± 26.34	0.005
	2 min	1.97 ± 0.29	2.06 ± 0.35	4.43 ± 3.71	0.005	1.89 ± 0.49	2.03 ± 0.53	7.50 ± 3.13	0.005
	3 min	1.91 ± 0.26	1.99 ± 0.28	3.75 ± 2.71	0.005	1.84 ± 0.48	1.95 ± 0.52	5.77 ± 3.75	0.005
	4 min	1.89 ± 0.23	1.94 ± 0.25	2.67 ± 2.45	0.007	1.83 ± 0.49	1.92 ± 0.54	4.22 ± 2.70	0.005
	5 min	1.87 ± 0.20	1.91 ± 0.20	2.19 ± 1.84	0.013	1.84 ± 0.51	1.90 ± 0.53	3.08 ± 1.59	0.005

#### The effect of two reconstruction algorithms on large and small positive lesions

**Fig. 4:**
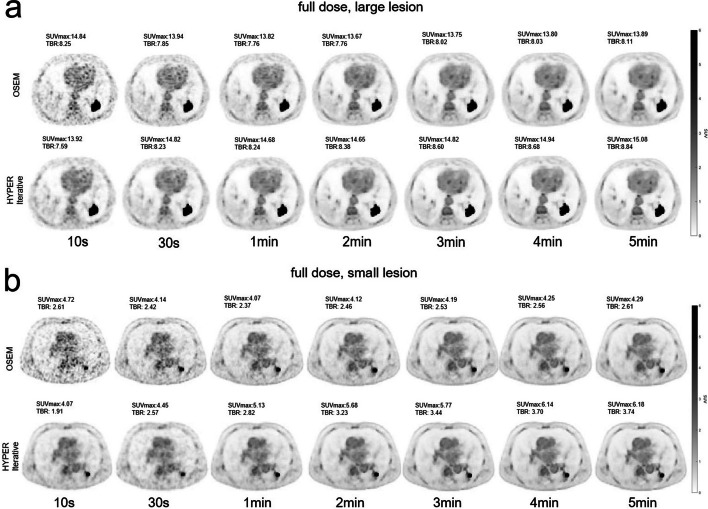
Transverse PET images of large and small lesions reconstructed by HYPER Iterative and OSEM with different scan times in two lung cancer patients injected with full-dose ^18^F-FDG. **a** ^18^F-FDG Transverse PET images reconstructed by HYPER Iterative and OSEM for a positive large lesion (diameter: 3 cm) with different scan times of 10 s, 30 s, 1 min, 2 min, 3 min, 4 min and 5 min, respectively. HYPER Iterative brings about 8–9% higher SUVmax and 8–9% higher TBR compared to OSEM reconstruction from 2 to 5 min. **b** ^18^F-FDG transverse PET images reconstructed by HYPER Iterative and OSEM for a positive small lesion (diameter: 8 mm) with different scan times of 10 s, 30 s, 1 min, 2 min, 3 min, 4 min and 5 min, respectively. HYPER Iterative brings about 24–45% higher SUVmax and 23–45% higher TBR compared to OSEM reconstruction from 1 to 5 min

**Fig. 5:**
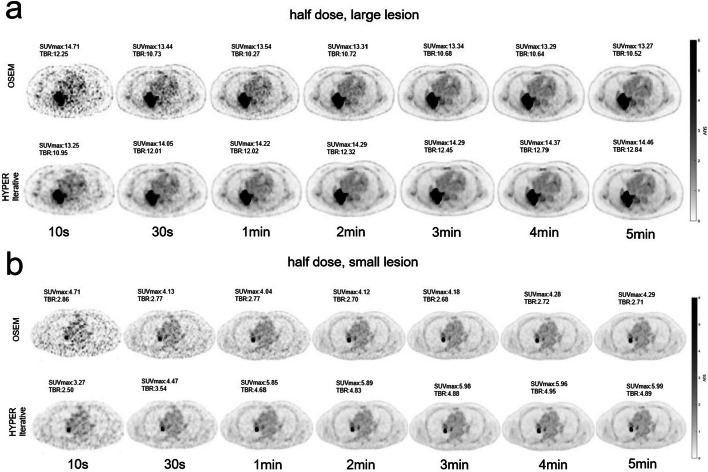
Transverse PET images of large and small lesions reconstructed by HYPER Iterative and OSEM with different scan times in two lung cancer patients injected with half-dose ^18^F-FDG. **a** ^18^F-FDG Transverse PET images reconstructed by HYPER Iterative and OSEM for a positive large lesion (diameter: 4 cm) with different scan times of 10 s, 30 s, 1 min, 2 min, 3 min, 4 min and 5 min, respectively. HYPER Iterative brings about 12–13% higher SUVmax and 18–23% higher TBR compared to OSEM reconstruction from 2 to 5 min. **b** ^18^F-FDG transverse PET images reconstructed by HYPER Iterative and OSEM for a positive small lesion (diameter: 7 mm) with different scan times of 10 s, 30 s, 1 min, 2 min, 3 min, 4 min and 5 min, respectively. HYPER Iterative brings about 61–75% higher SUVmax and 77–94% higher TBR compared to OSEM reconstruction from 1 to 5 min

**Fig. 6:**
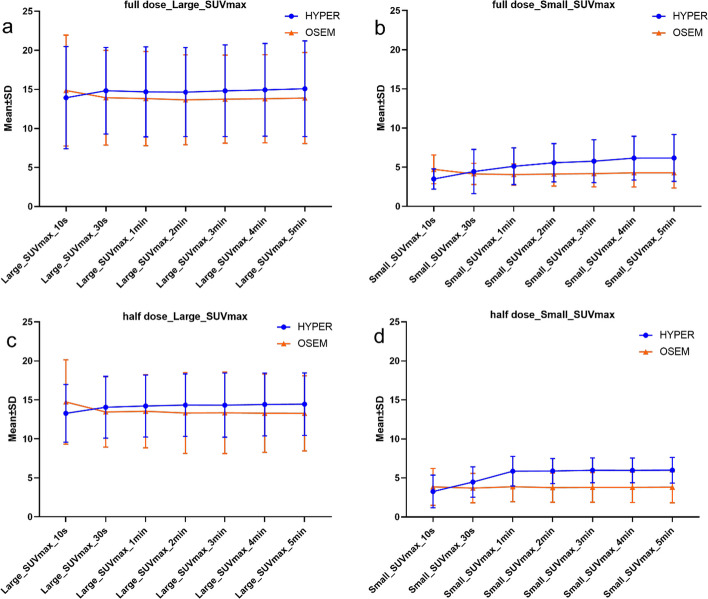
The SUVmax of positive large and positive small lesions in full-dose and half-dose groups reconstructed by OSEM and HYPER Iterative algorithms at different scan times

**Fig. 7:**
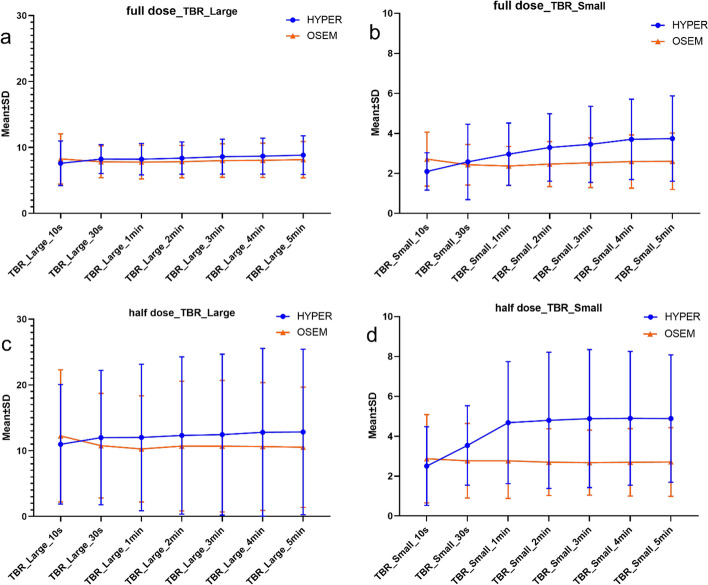
The TBR of positive large and small lesions in full-dose and half-dose groups reconstructed by OSEM and HYPER Iterative algorithms at different scan times

For large positive lesions ≥ 2.0 cm, the SUVmax reconstructed by the HYPER Iterative in the full dose was significantly higher than that reconstructed by OSEM with a scan time from 2 to 5 min (*P* < 0.05), which was approximately 9.03% higher at 5 min, as shown in Figs. 4, 6 and Table 6. A similar trend can be observed for the half dose, which was up to 12.52% higher at 5 min (Figs. 5, 6 and Table 6). However, no significant difference in SUVmax between the two reconstruction algorithms was observed within 1 min (*P* > 0.05), either for the full dose or the half dose. For small positive lesions ≤ 10 mm, the SUVmax of HYPER Iterative reconstruction in the full dose was higher (*P* < 0.05) than that of OSEM for a scan time from 1 to 5 min, which was 45.21% higher for a scan time of 5 min. In the half dose, this difference was increased up to 74.96% at 5 min (*P* < 0.05). No significant difference in SUVmax for small lesions was found between the two reconstruction algorithms within 30 s (*P* > 0.05) in either the full-dose or half-dose groups. Similar to SUVmax, the TBR of the large lesions with the HYPER Iterative was higher than that with OSEM for scan times from 2 to 5 min (*P* < 0.05). TBR by HYPER Iterative was 8.69% higher for the full dose at 5 min and 23.39% higher for the half dose at 5 min compared with that by OSEM reconstruction (Fig. 7) (Table 7). However, no significant difference between the two reconstruction algorithms was observed within 1 min (*P* > 0.05). For small lesions, the TBR of small lesions reconstructed by HYPER Iterative was higher than that reconstructed by OSEM within 1 min to 5 min (*P* < 0.05). At 5 min, the HYPER Iterative reconstruction was 44.91% and 93.73% higher in the full-dose and half-dose groups, respectively (Fig. 7 and Table 7). However, no significant difference was found between the two reconstruction algorithms within 30 s (*P* > 0.05).

**Table 6:** SUVmax of large and small lesions in different scan times and injected dose reconstructed by HYPER Iterative and OSEM

Lesion	Time	Full dose group (*n* = 10)				Half dose group (*n* = 10)			
		HYPER Iterative	OSEM	Difference (%)	*P*	HYPER Iterative	OSEM	Difference (%)	*P*
SUVmax (large lesion)	10 s	13.93 ± 6.55	14.85 ± 7.10	4.51 ± 6.53	0.074	13.28 ± 3.69	14.73 ± 5.40	4.15 ± 30.48	0.139
	30 s	14.83 ± 5.54	13.94 ± 6.07	8.83 ± 15.03	0.139	14.06 ± 3.96	13.45 ± 4.51	6.99 ± 20.83	0.333
	1 min	14.69 ± 5.76	13.82 ± 6.04	8.07 ± 15.01	0.114	14.22 ± 3.98	13.54 ± 4.69	7.92 ± 20.44	0.203
	2 min	14.65 ± 5.70	13.66 ± 5.76	8.37 ± 11.80	0.017	14.29 ± 3.99	13.32 ± 5.19	12.10 ± 20.79	0.047
	3 min	14.82 ± 5.87	13.75 ± 5.64	8.40 ± 10.57	0.009	14.28 ± 4.11	13.34 ± 5.23	11.77 ± 20.66	0.047
	4 min	14.94 ± 5.94	13.80 ± 5.64	8.76 ± 10.54	0.013	14.39 ± 4.02	13.29 ± 5.01	12.76 ± 20.87	0.047
	5 min	15.08 ± 6.13	13.89 ± 5.83	9.03 ± 10.61	0.013	14.45 ± 4.00	13.27 ± 4.82	12.52 ± 19.20	0.047
SUVmax (Small lesion)	10 s	3.59 ± 1.30	4.73 ± 1.84	10.39 ± 47.25	0.333	3.26 ± 2.09	3.85 ± 2.35	4.84 ± 68.96	0.139
	30 s	4.45 ± 2.82	4.14 ± 1.35	1.82 ± 31.29	0.878	4.47 ± 1.94	3.70 ± 1.89	28.21 ± 36.80	0.139
	1 min	5.12 ± 2.35	4.06 ± 1.36	24.19 ± 28.95	0.028	5.86 ± 1.90	3.86 ± 1.91	61.46 ± 29.27	0.005
	2 min	5.57 ± 2.45	4.13 ± 1.53	33.30 ± 22.22	0.007	5.89 ± 1.61	3.75 ± 1.87	71.70 ± 46.61	0.005
	3 min	5.77 ± 2.74	4.19 ± 1.69	36.11 ± 20.51	0.005	5.98 ± 1.59	3.78 ± 1.89	73.96 ± 47.79	0.005
	4 min	6.16 ± 2.79	4.29 ± 1.81	44.17 ± 20.94	0.005	5.96 ± 1.59	3.79 ± 1.95	74.27 ± 47.42	0.005
	5 min	6.18 ± 2.99	4.29 ± 1.94	45.21 ± 22.57	0.005	5.99 ± 1.64	3.81 ± 1.99	74.96 ± 48.77	0.005

**Table 7:** TBR of large and small lesions in different scan times and injected dose reconstructed by HYPER Iterative and OSEM

Lesion	Time	Full dose group (*n* = 10)				Half dose group (*n* = 10)			
		HYPER Iterative	OSEM	Difference (%)	*P*	HYPER Iterative	OSEM	Difference (%)	*P*
TBR (large lesion)	10 s	7.60 ± 3.37	8.27 ± 3.81	6.77 ± 9.00	0.074	10.97 ± 9.09	12.25 ± 10.05	2.38 ± 34.91	0.203
	30 s	8.24 ± 2.19	7.83 ± 2.41	6.58 ± 11.52	0.169	12.00 ± 10.22	10.76 ± 7.96	11.17 ± 24.75	0.508
	1 min	8.22 ± 2.39	7.78 ± 2.57	6.99 ± 13.69	0.241	12.02 ± 11.15	10.27 ± 8.07	15.15 ± 23.62	0.074
	2 min	8.39 ± 2.45	7.85 ± 2.46	7.55 ± 10.95	0.028	12.32 ± 11.96	10.70 ± 9.87	17.63 ± 25.59	0.037
	3 min	8.60 ± 2.64	8.01 ± 2.53	7.67 ± 9.73	0.013	12.45 ± 12.23	10.68 ± 10.01	19.40 ± 29.94	0.013
	4 min	8.68 ± 2.73	8.05 ± 2.60	8.31 ± 9.85	0.013	12.80 ± 12.76	10.64 ± 9.73	23.13 ± 35.54	0.047
	5 min	8.84 ± 2.93	8.15 ± 2.75	8.69 ± 9.72	0.013	12.84 ± 12.58	10.53 ± 9.15	23.39 ± 34.15	0.028
TBR (small lesion)	10 s	2.10 ± 0.93	2.72 ± 1.35	7.50 ± 23.64	0.074	2.50 ± 1.97	2.87 ± 2.22	4.15 ± 58.74	0.445
	30 s	2.58 ± 1.88	2.43 ± 1.01	0.23 ± 31.59	0.878	3.54 ± 1.99	2.77 ± 1.87	38.04 ± 58.65	0.169
	1 min	2.96 ± 1.55	2.37 ± 0.97	23.18 ± 29.32	0.028	4.68 ± 3.06	2.77 ± 1.89	77.21 ± 63.31	0.005
	2 min	3.29 ± 1.69	2.47 ± 1.13	32.37 ± 22.13	0.007	4.80 ± 3.42	2.70 ± 1.67	85.43 ± 77.43	0.005
	3 min	3.45 ± 1.90	2.53 ± 1.24	35.27 ± 20.43	0.005	4.88 ± 3.46	2.68 ± 1.63	89.92 ± 77.90	0.005
	4 min	3.70 ± 2.01	2.60 ± 1.33	43.69 ± 21.22	0.005	4.90 ± 3.35	2.70 ± 1.69	92.26 ± 75.32	0.005
	5 min	3.74 ± 2.13	2.60 ± 1.41	44.91 ± 23.00	0.005	4.89 ± 3.19	2.71 ± 1.72	93.73 ± 76.68	0.005

For the MTV of the lesions, two reconstruction algorithms did not bring about a significant difference (all *P* > 0.05), not only in the full-dose group, but also in the half-dose group at each time point (Additional file 1: Table S1).

## Discussion

Benefiting from the extralong-axial FOV and its 40 times higher sensitivity compared to conventional PET/CT, uEXPLORER is able to complete a total-body PET/CT scan in a very short time [16, 17]. Our study confirmed that high-quality total-body PET can be obtained within a very short scan time using uEXPLORER PET/CT, especially when using HYPER Iterative reconstruction, which resulted in a high kappa coefficient of visual evaluation of the image quality between the two readers in the two groups. When HYPER Iterative reconstruction was used, only 1 min of acquisition was needed to generate a high-quality image for patients injected with a full dose of ^18^F-FDG or 2 min for a half dose. Compared with the HYPER Iterative, OSEM requires a slightly longer time to obtain comparable high-quality images, with 2 min for the full dose and 3 min for the half dose. Similar results have been reported, where 4 min scanning using uEXPLORER PET/CT injected with a half-dose ^18^F-FDG and reconstructed by OSEM could achieve good image quality (scores, 4.9 ± 0.2), which was better than that of conventional PET/CT with a clinical routine full-dose ^18^F-FDG in lung cancer [18]. Similarly, it was also reported that the long-axial field-of-view (LAFOV) Biograph Vision Quadra PET/CT could produce images of comparable quality and lesion quantification under 2 min compared to a standard-axial field-of-view (SAFOV) Biograph Vision 600 PET/CT (16 min for equivalent FOV coverage) [28]. Compared to the 20–30 min acquisition for the conventional PET/CT whole-body [29–32], the efficiency of uEXPLORER PET/CT is dramatically improved. This ultrahigh imaging efficiency brings great benefits to the clinic, including (1) easier adaptation for patients with physical weakness, unbearable pain and claustrophobia or difficulty cooperating; (2) higher daily throughput (60–80 patients/day) to meet high PET/CT imaging need; and (3) reducing the radioactive exposure by decreasing the injected dose and slightly prolonging the scan time (from 1 to 2 min), which is very important for adolescent patients who suffer from lymphoma and need multiple PET/CT imaging studies [33–37].

The present study has shown that high PET image quality can be obtained in a shorter time with the HYPER Iterative compared with OSEM, which is similar to the findings by Sui [25]. Their research showed that HYPER Iterative reconstructions could provide better lesion visibility and noise reduction than OSEM reconstruction injected with ultralow doses of ^18^F-FDG (0.37 MBq/kg). The difference in the image quality between these two algorithms is more obvious with a very short scan time, i.e. 10 s and 30 s. In a short acquisition time (such as 10 s), the acquired total count rate is very low, which will generate large statistical fluctuations and noise. OSEM reconstruction cannot suppress such noise, leading to very poor image quality and a conspicuously high false SUVmax. In contrast, the HYPER Iterative can significantly suppress the noise by making use of its penalty function to control for any excessive image noise. As a result, HYPER Iterative can obtain a much higher quality PET image even with a very short acquisition time (such as 10 s) compared to OSEM, as illustrated in this study. The measured SUVmax in the HYPER Iterative reconstructed images was only slightly influenced by the noise. Therefore, the SUVmax at 10 s was much higher after OSEM reconstruction than after HYPER Iterative reconstruction. As the acquisition time increased, the noise decreased due to the greater number of counts, and its impact decreased. In contrast, the reconstruction accuracy of the HYPER Iterative contributed to a high SUVmax and a higher lesion contrast after 10 s. The advantage of HYPER Iterative reconstruction is of utmost importance for dynamic imaging acquisition because it always needs to assign very short intervals, such as 10 s or 30 s, to observe the rapid change in radioactivity distribution, especially during the early phase [38, 39]. It is also useful for patients who receive a very low injected dose, and the total-body scan needs to be completed in a short time [40, 41].

The present study demonstrated that HYPER Iterative reconstruction is useful for visualizing positive lesions. For positive lesions, the detection accuracy of PET/CT depends on two main parameters: the uptake of the tracer by the lesion and the signal contrast, such as TBR. The higher the SUVmax and TBR are, the more clearly the lesion can be depicted and more easily detected [42–44]. The results showed that the SUVmax and TBR of lesions obtained by the HYPER Iterative were higher than those obtained by OSEM. For large lesions, HYPER Iterative brings up to 9.03% higher SUVmax and up to 8.69% higher TBR compared to OSEM in the full-dose group. In the half-dose group, up to 12.52% higher SUVmax and up to 23.39% higher TBR were obtained by HYPER Iterative reconstruction than by OSEM. The advantage of HYPER Iterative on small lesion detection was more obvious. The SUVmax and TBR generated by HYPER Iterative were 45.21% and 44.91% higher in the full-dose group and up to 74.96% and 93.73% higher in the half-dose group than those generated by OSEM. The high sensitivity of detecting small lesions is important not only for diagnosing early cancer but also for accurate staging [45–47]. A higher SUVmax and TBR in small lesions obtained by HYPER Iterative reconstruction will improve the diagnostic ability of PET/CT for malignant tumours. HYPER Iterative reconstruction may alleviate the influence of the partial volume effect on visualizing small lesions compared with OSEM reconstruction. In the present study, the SUVmax and TBR of the small lesions on HYPER Iterative seemed to be count rate dependent, so they were stable when the images had high enough count rates after 1–2 min of acquisition, and the advantage of HYPER Iterative for visualizing small lesions was exhibited.

In the present study, compared to OSEM reconstruction, a higher SD of SUVmax for small lesions in the full-dose group on HYPER Iterative was observed, which was due to significant increase in SUVmax in one lesion when using HYPER Iterative reconstruction. A similar phenomenon occurred in the TBR of the smaller lesions in the half-dose group on HYPER Iterative, which was also caused by a significant increase in the TBR of one lesion when using HYPER Iterative reconstruction.

There are some limitations of this study: (1) the whole scan time for each patient was relatively short (5 min), and the difference between the two reconstruction algorithms for image quality and lesion visualization for longer scan times was not included. (2) The sample size was too small, and more research is needed to confirm the findings of this work.

## Conclusion

Our study demonstrates the excellent imaging performance of uEXPLORER PET/CT for total-body imaging, which can be acquired with high quality within a very short time (1–2 min). Compared with OSEM, HYPER Iterative can obtain a higher quality PET image in a shorter scan time. A 1 min scan with a full dose and a 2 min scan with a half dose are ideal for clinical diagnosis using the HYPER Iterative, while a 2 min scan with a full dose and a 3 min scan with a half dose are required for OSEM reconstruction. A higher SUVmax and TBR can be obtained using the HYPER Iterative compared to OSEM, especially for small lesions. Therefore, for quantification of small lesions, the HYPER Iterative is preferred, especially for the half-dose ^18^F-FDG scenario. More research is warranted to confirm our findings.

### Supplementary Information

**Additional file 1. Table 1**: The MTV of large and small lesions in the full-dose group and the half-dose group at each time point reconstructed by the two algorithms.

## Data Availability

The data that support the findings of this study are available from the corresponding author upon reasonable request.
