# Supplementary material for: A proper protocol for routine 18F-FDG uEXPLORER total-body PET/CT scans
Source: EJNMMI Phys. 2023 Sep 11;10:51. doi: 10.1186/s40658-023-00573-4 (PMC10495295; doi:10.1186/s40658-023-00573-4)
Supplement: Supplementary file 1 — Additional file 1. Table 1: The MTV of large and small lesions in the full-dose group and the half-dose group at each time point reconstructed by the two algorithms. [file 40658_2023_573_MOESM1_ESM.docx]

Supplementary Materials

Table 1 The MTV of large and small lesions in the full-dose group and the half-dose group at each time point reconstructed by the two algorithms

| **Lesion** | **Time** | **Full dose group (n=10)** | | | **Half dose group (n=10)** | | |
| --- | --- | --- | --- | --- | --- | --- | --- |
|  |  | **HYPER Iterative** | **OSEM** | ***P*** | **HYPER Iterative** | **OSEM** | ***P*** |
| large lesion_MTV (mm^3^) | 10 s | 2372.9±803.1 | 2516.3±926.3 | 0.1 | 2024.9±772.9 | 2278.7±850.4 | 0.1 |
|  | 30 s | 2465.9±847.3 | 2477.1±867.9 | 0.6 | 2425.1±861.4 | 2273.0±813.8 | 0.3 |
|  | 1 min | 2516.6±886.5 | 2485.6±875.4 | 0.1 | 2492.8±930.6 | 2287.0±781.0 | 0.4 |
|  | 2 min | 2553.2±909.0 | 2505.3±896.8 | 0.1 | 2490.0±895.2 | 2160.2±756.8 | 0.2 |
|  | 3 min | 2573.0±915.6 | 2516.6±868.8 | 0.1 | 2475.9±863.1 | 2233.5±803.0 | 0.3 |
|  | 4 min | 2587.0±911.2 | 2530.7±864.1 | 0.1 | 2335.1±812.5 | 2284.2±780.5 | 0.3 |
|  | 5 min | 2584.2±913.8 | 2522.2±860.1 | 0.1 | 2557.6±898.2 | 2304.0±732.2 | 0.3 |
| small lesion_MTV (mm^3^) | 10 s | 160.6±54.9 | 174.7±56.1 | 0.1 | 98.6±30.4 | 98.6±30.4 | 1.0 |
|  | 30 s | 177.6±59.5 | 166.3±68.3 | 0.1 | 98.6±30.4 | 98.6±30.4 | 1.0 |
|  | 1 min | 180.4±62.6 | 166.3±60.1 | 0.1 | 98.6±30.4 | 95.8±27.2 | 0.3 |
|  | 2 min | 181.3±65.6 | 157.8±70.6 | 0.1 | 101.5±35.6 | 98.6±30.4 | 0.3 |
|  | 3 min | 182.2±62.1 | 160.6±69.1 | 0.1 | 104.2±42.1 | 101.4±35.6 | 0.3 |
|  | 4 min | 183.5±59.1 | 166.3±64.3 | 0.1 | 104.2±42.1 | 101.4±35.6 | 0.3 |
|  | 5 min | 184.3±62.1 | 157.8±64.0 | 0.1 | 104.2±42.1 | 104.2±42.1 | 1.0 |
